# Co-Infection by Hepatitis C Virus in HIV-Infected Patients in Southern Brazil: Genotype Distribution and Clinical Correlates

**DOI:** 10.1371/journal.pone.0010494

**Published:** 2010-05-05

**Authors:** Fernando H. Wolff, Sandra C. Fuchs, Nêmora N. T. Barcellos, Paulo Ricardo de Alencastro, Maria Letícia R. Ikeda, Ajácio B. M. Brandão, Maicon Falavigna, Flávio D. Fuchs

**Affiliations:** 1 National Institute of Science and Technology for Health Technology Assessment (IATS)–CNPq, Porto Alegre, Rio Grande do Sul, Brazil; 2 Post-graduate Program in Medical Sciences, School of Medicine, Federal University of Rio Grande do Sul, Porto Alegre, Rio Grande do Sul, Brazil; 3 Post-Graduate Program in Epidemiology, School of Medicine, Federal University of Rio Grande do Sul, Porto Alegre, Rio Grande do Sul, Brazil; 4 Therapeutic Attention Service, Sanatório Partenon Hospital, Porto Alegre, Rio Grande do Sul, Brazil; 5 School of Medicine, Federal University of Health Sciences of Porto Alegre, Porto Alegre, Rio Grande do Sul, Brazil; 6 Centre for AIDS Studies of Rio Grande do Sul (Centro de Estudos em AIDS do Rio Grande do Sul - CEARGS), Porto Alegre, Rio Grande do Sul, Brazil; 7 Division of Cardiology, Hospital de Clínicas de Porto Alegre, Porto Alegre, Rio Grande do Sul, Brazil; Saint Louis University, United States of America

## Abstract

**Background:**

Prevalence rates of Hepatitis C Virus (HCV) co-infection, the distribution of HCV genotypes, and the frequency of spontaneous resolution of hepatitis C in patients infected with the Human Immunodeficiency Virus (HIV) have a worldwide disparity. The purpose of this study is to investigate the prevalence of HCV antibodies (anti-HCV) in patients with HIV, the proportion and correlates of infection by different HCV genotypes, and rates of spontaneous resolution of HCV infection.

**Methods:**

A cross-sectional study was conducted among 1143 HIV patients under follow-up in a HIV/AIDS outpatient reference center of the Brazilian public health system. From 357 anti-HCV positive patients, a consecutive sample of 227 individuals HCV treatment-naïve was interviewed and 207 was tested for HCV-RNA and genotypes.

**Results:**

Anti-HCV was detected in 357 patients (31.2%). HCV-RNA was undetectable in 16.4% of 207 anti-HCV positive individuals. Genotype 1 was diagnosed in 81.5% of the sample, genotype 2 in 1.7% and genotype 3 in 16.2%. Male gender was the unique characteristic associated with higher prevalence of genotype 1 HCV.

**Conclusions:**

Co-infection by HCV is frequent among patients with HIV in our State, and it is particularly high the infection by HCV genotype 1. Further investigation is necessary to explain the important regional variation in the proportion of infection by the different HCV genotypes and to better understand rates of spontaneous HCV clearance.

## Introduction

It has been estimated that 38.6 million people are infected by the human immunodeficiency virus (HIV) worldwide, and 4–5 million are also infected with hepatitis C virus (HCV) [1]. In Brazil, about 0.5% of the adult population is infected with HIV [1] and among those attending the public health services, 16% HIV-HCV co-infection rate had been reported in outpatient care centers [2], [3], 42% in HIV-HCV anonymous testing centers [4], and 54% for patients HIV infected in infectious diseases clinics [5].

The survival of HIV infected patients has markedly improved since the introduction of highly active antiretroviral treatment (HAART). [6] While treatment of HIV-HCV co-infected patients has lower rates of sustained virological response (SVR), it might also be complicated by adverse effects and drug interaction. Therefore, the selection of patients with a favorable risk/benefit ratio to respond to treatment is necessary. The best predictor of treatment effectiveness is the HCV genotype and, consequently, it becomes a key factor to support therapeutic decisions. [7]–[11] Besides the treatment issues, geographical distribution of HCV genotypes might help to elucidate new routes of infection among HIV infected patients.

This study investigated the prevalence of HCV antibodies (anti-HCV) in patients with HIV, the proportion and correlates of infection by different HCV genotypes, and rates of undetectable HCV-RNA in anti-HCV positive patients treatment-naïve (hepatitis C spontaneous resolution).

## Methods

### Study design

This cross-sectional study was conducted in a public reference center for HIV testing and treatment in Porto Alegre, southern Brazil. Patients were referred by primary care doctors or anonymous testing centers.

The routine protocol for evaluation of patients included complete medical history and physical examination, basic hematological and biochemical tests, toxoplasmosis screening test, PPD skin test, chest X-ray, CD4, HIV viral load, hepatitis B surface antigen (HBsAg) and hepatitis C antibodies (anti-HCV). Anti-HCV was performed using third-generation enzyme-linked immunosorbent assays (ELISA-3 Hepanostika® - BioMerrie).

### Participants

A registry of 3490 patients with HIV infection was launched in 1996 in our center, comprising all patients with at least one appointment with the infectious diseases doctor. All records were retrieved and the follow-up of surviving participants was updated until 2006. The eligibility criteria included patients aged 18 years or older, who had at least one appointment in the previous 12 months and were co-infected by HCV. The final sample comprised 1143 HIV patients actively under follow-up.

All patients with an anti-HCV positive test were consecutively interviewed from March-2005 to September-2006, during the routine visit to the attending physician. Patients who did not complete the study protocol at that day were re-scheduled to prevent losses.

### Data collection

Interviewers were trained and certified to conduct the data collection, and quality control was carried out at random by one of the investigators. Patients were interviewed using a standardized questionnaire, which included questions pertaining demographic (gender, age, skin color) and behavioral (alcohol consumption, tattooing, sharing personal hygiene objects - tooth brushes, shaving blades, cuticle nippers -, acupuncture) characteristics, socioeconomic status (family income, schooling), medical history (blood transfusion, accidental exposure to biological material), sexual behavior (age at first sexual intercourse, sexual orientation, anal sex, use of condom, number of sexual partners), and use of illicit drugs (injecting drugs, snorting cocaine, crack cocaine, marijuana, solvents/inhalants). Variables associated with HIV infection (CD4 lymphocyte count, CD4/CD8 ratio, use of antiretroviral drugs, duration of HIV diagnosis, opportunistic infections) were also investigated.

After the interview, blood samples were collected and processed for HCV RNA detection and HCV genotype determination. The laboratory was certified by the Brazilian Society of Clinical Pathology and Laboratory Medicine.

### HCV RNA detection and genotyping

The real-time polymerase chain reaction (RT-PCR) technique was used for the qualitative detection of HCV RNA as described elsewhere [12]–[14]. Genotypes were identified using the restriction fragment length polymorphism (RFLP) [15], adapted as described elsewhere [16]. The lower limit of detection of the test is 50 UI/mL. All tests were performed in duplicate, and reliability of HCV RNA was tested in a random sampling of 10% of the patients. Two thirds of undetectable HCV-RNA patients were retested.

### Study variables

The main study outcomes were the detection of HCV antibodies, defined as a positive result in the anti-HCV test, and the prevalence of HCV infection according to the genotypes. In addition, we performed an exploratory analysis on characteristics associated with spontaneous resolution of HCV infection, characterized as an undetectable HCV-RNA in an anti-HCV positive patients who have never been submitted to an interferon based therapy.

The self-report of skin color was categorized into white or non-white. Schooling was measured by the number of years at school. Heterosexual orientation was established for those who reported sexual intercourse with partners of the opposite sex; man who have sex with men, as well as women who have sex with women, were categorized as homosexuals, and bisexuals were defined when they reported having sex with men and women [17]. Consumption of alcoholic beverages, smoking and use of illicit drugs were investigated using standardized questions. CD4, CD8 counts and HIV viral load, determined closest to the appointment date, were used in the analysis. Antiretroviral treatment was defined as taking any medicine at the time of the interview. The duration of HIV infection was calculated from the date of the diagnosis to the date of the interview as reported by the patient and confirmed with medical records.

### Statistical analysis

Data were entered in duplicate and checked for consistency and reliability using the Epinfo 3.3.2 software. The Statistical Package for the Social Sciences (SPSS, version 14.0, Chicago, Il, USA) was used for statistical analyses. Pearson chi-square test and analysis of variance were used to analyze characteristics associated with HCV RNA and genotypes. P value <0.05 was considered statistically significant and a trend toward association was established for p value >0.05 and <0.15. Point prevalence and 95% confidence interval (CI) were presented for HIV-HCV coinfection, HCV RNA and genotypes. The Institutional Review Board and Ethical Committee approved the protocol, and all participants gave informed consent.

## Results

**Figure 1:**
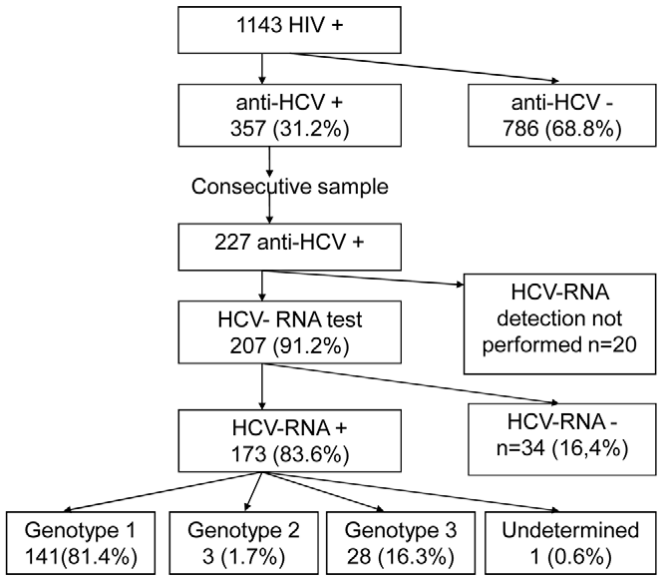
HCV-HIV prevalence and genotype distribution. Flowchart of inclusion in the different stages of the study, and patient's prevalence of anti-HCV positive, HCV-RNA positive and HCV genotypes.

Anti-HCV testing was performed in 1143 patients. A positive anti-HCV test was detected for 357 (31.2%) individuals. From March-2005 to September-2006, all patients who went to the routine evaluation were interviewed, resulting in the enrollment of a consecutive sample of 227 co-infected patients. RNA HCV testing was performed in 207 patients (Figure 1), as 20 patients didn't come to the blood collecting laboratory to be tested. The comparison between patients with and without HCV-RNA testing results available did not reveal differences regarding demographic characteristics.

Table 1 shows that most participants were men, aged on average 40 years, had white skin color, injecting cocaine users, and approximately 19% were bisexuals.

**Table 1:** Characteristics of HIV infected patients with anti-HCV positive test (N = 227).

		N (%) or mean ±SD
Male gender (%)		143 (63%)
Age (years)		40.3±8.7
White skin color		129 (56.8)
Schooling (years)		6,7±3.4
Sexual orientation		
	Heterosexual	97 (76.3)
	Homosexual	6 (4.8)
	Bisexual	24 (18.9)
Transfusion before 1993		24 (10.6)
Use of illicit drugs		
	Marijuana	148 (65.2)
	Inhaled cocaine	138 (60.8)
	Injecting	127 (55.9)
	Crack cocaine	62 (27.3)
CD4 (cells/mm^3^)		387±217
Duration of HIV diagnosis (years)		6,0±4.5

### Prevalence of HIV-HCV coinfection and HCV genotypes

Figure 1 shows that among 207 HIV-HCV co-infected patients, according to the anti-HCV test, the infection was confirmed based on a positive HCV-RNA for 173 (83.6%) participants. No of the included patients had been submitted to antiviral therapy for HCV.

Genotype 1 HCV was the most prevalent (81.5%; 95%CI 75.7–87.3), followed by genotype 3 (16.2%; 95%CI 10.7–21.7), and only two cases had genotype 2 (1.7%; 95%CI 0–3.6). Genotypes 4, 5 and 6 HCV were not detected and virus C genotype could not be determined in one patient.

### Characteristics associated with HCV genotypes

Table 2 shows that most characteristics were not significantly associated to the genotype, except higher prevalence of genotype 1 among men. There was a trend toward an association with transfusions in the past. Patients with genotype 1 were more likely to be on antiretroviral treatment and have longer duration of HIV infection than genotype 3. Also, a trend for a lower HIV viral load in patients infected with HCV genotype 3 compared to 1 was found. (Table 3)

**Table 2:** Characteristics of patients coinfected with HIV-HCV according to HCV genotype [N (%) or mean ±SD].

		Total*	Genotype 1	Genotype 3	*P*
		*N = 173*	*N = 141*	*N = 28*	*Value*
Male sex		111 (64.2)	97 (68.8)	12 (42.9)	*0.009*
Age (years)		40.1±8.9	40.1±8.7	40.3±10.2	*0.9*
White skin color		96 (55.5)	78 (55.3)	18 (64.3)	*0.4*
Schooling (years)		6.6±3.5	6.6±3.4	6.7±3.6	*0.3*
Sexual orientation					*0.6*
	Heterosexual	131 (75.6)	104 (73.8)	23 (82.1)	
	Homosexual	6 (3.5)	5 (3.5)	1 (3.6)	
	Bisexual	36 (20.9)	32 (22.7)	4 (14.3)	
Transfusion before 1993		20 (11.6)	19 (13.5)	1 (3.6)	*0.14*
Illicit drug use					
	Marijuana	119 (68.8)	102 (72.3)	15 (53.6)	*0.5*
	Snorting cocaine	112 (64.7)	94 (66.7)	16 (57.1)	*0.3*
	Injecting	103 (59.5)	86 (61.0)	15 (53.6)	*0.5*
	Crack	50 (28.9)	40 (28.4)	8 (28.6)	*1.0*

*total number of patients includes 3 cases of genotype 2 and 1 of undetermined genotype.

**Table 3:** Characteristics associated with HIV according to HCV genotype [N (%) or mean±SD].

		Total*	Genotype 1	Genotype 3	P value
		N = 173	N = 141	N = 28	
CD4 (cells/µl)					*0.9*
	≤200	28 (15.9)	23 (16.3)	4 (14.3)	
	201–350	63 (36.5)	52 (36.9)	9 (32.1)	
	>350	82 (47.6)	65 (46.1)	14 (50.0)	
CD8 (cells/µl)		1118±903	1147±980	955±320	*0.6*
CD4/CD8 ratio		0.48±0.47	0.49±0.50	0.48±0.29	*0.9*
HIV viral load <50 copies/ml		69 (39.9)	60 (42.6)	8 (28.6)	*0.2*
Use of antiretroviral drug		141 (81.5)	119 (84.4)	19 (67.9)	*0.04*
Time since HIV diagnosis (years)		5.4±3.9	5.6±3.9	4.5±3.3	*0.1*

*total number of patients includes 3 cases of genotype 2 and 1 of undetermined genotype.

### Characteristics of HCV RNA negative patients

Thirty four patients (16.9%) had negative HCV RNA test. Undetectable HCV RNA was more frequent among those who 102 individual who reported sharing of personal hygiene objects (22.0% *vs.*10.8%; P = 0.03). The prevalence of undetectable HCV RNA levels was high among homosexuals (45.5%), intermediate between heterosexuals (16.7%) and reduced for bisexuals (7.7%) (P = 0.01).

## Discussion

This study detected high prevalence of individuals co-infected with HCV (31.2%) among patients with HIV in our State. It was similar to the described in the United States (31–36%) [18], [19] for patients under follow-up at infectious diseases clinics and to that reported for volunteers of a multicenter trial conducted mainly in Europe (31%) [20]. This prevalence was higher than those reported in public health services for HIV patients from the North (16%) and Southeast (18%) of Brazil [3]. Since HIV and HCV are transmitted by large or repeated exposures to infected blood, the difference in prevalence of co-infection among regions or countries might vary according to the distribution of risk factors, noticeably, injecting drug use [21]. In countries with no remarkable variation on IDU prevalence across regions, other risk factors such as sharing personal hygiene objects may play a major role [22].

The genotype 1 prevalence of 81.4% was higher than previously described among individuals infected (50.9%) [23] and non-infected with HIV (50%) in Brazil. [23]–[25] However, participants of those studies were referred to the specialist for evaluation and treatment of hepatitis C. Differently from studies in which an evaluation by a gastroenterologist or a clinical trial are the source of enrollment, patients from our study were withdraw from a broad spectrum of HIV infected patients, and this may account for a sample of HIV-HCV coinfected individuals that better represents the true genotype distribution in our setting. Worldwide, genotype 1 prevalence in patients co-infected by the HIV-HCV also showed a large variation: from 26% in China [26], to 53 to 75% in Spain [27], [28], and to 87% in United States [29]. The high prevalence of genotype 1 has been associated to the transmission of HCV by injecting drug use [21] which accounted for approximately 22-fold increase the risk of co-infection, in this population. [22] Genotypes 4, 5 and 6 HCV were not detected, which is in accordance with other reports. [23]–[25], [30]

The only demographic variable associated to HCV genotype was male sex. The prevalence of males was greater among genotype 1 carriers than among genotype 3 patients. The reasons for this difference are not completely understood, and, our sample are not enough large (97 males with genotype 1 and 12 with genotype 3) to explore this finding in a more detailed basis. Small epidemiological differences that didn't reach statistical significance in our study, such as rates of illicit drug use, blood transfusion, and sexual behavior, may be the link to this finding when taken together. We have no reason to believe males or females are biologically prone to contract a specific HCV genotype. Instead, we believe that epidemiological circumstances exposed man or woman to the genotype they became infected. Our data suggest small differences in characteristics associated with HIV according to HCV genotypes. The only data witch presented statistically significant difference was the higher antiretroviral use among HCV genotype 1 compared to genotype 3 patients. The trends of a higher time since HIV diagnosis and higher prevalence of HIV viral load <50 copies/mL in genotype 1 versus 3 patients are probably related one to another, since the longer the time since HIV diagnosis, greater are the chances of antiretroviral treatment and, once one treatment, greater the chances of achieving undetectable HIV viral load. Data regarding association between HCV genotypes and HIV characteristics are scarce. Our data are in accordance with data already published by Soriano et al. [31] The EuroSIDA study authors reported higher antiretroviral use among HCV genotype 1 than genotype 3 infected patients. They also found no CD4 cell count and HIV viral load difference according to HCV genotype. [31]

Few studies have investigated systematically the prevalence of co-infection by HCV-RNA in patients with HIV infection. In the EuroSIDA less than 10% of the individuals with anti-HCV positive results had the infection confirmed by HCV-RNA detection [20]. In this study, high rate of patients (91.2%) were tested for HCV-RNA using real time-PCR technique, and a sample of negative and positive tests (40%) were repeated in order to reassure reproducibility. In a multi-center study, conducted in Europe and Australia, only 63% of patients with anti-HCV positive results underwent to HCV-RNA test, and 82% had a positive result [32]. None of the patients included in the study had been exposed to HCV treatment previously to the interview, and a false negative PCR result is unlikely since the HCV-RNA detection technique has high sensitivity [33] and all samples were processed in duplicates. Therefore, it is likely that participants with anti-HCV without detectable HCV viremia (16%) had spontaneous resolution of the infection. Several factors have been associated with spontaneous resolution of HCV viremia, such as cell immune responses [34]–[36], Asian or African ethnicity [37], and parenteral exposure to the virus [38]. Spontaneous resolution would be lower in individuals co-infected with HIV. [34] This study was not designed with enough power to investigate factors associated to spontaneous resolution. We hypothesize that individuals who have been exposed only to small amount of inoculum, such as the transmission by sharing personal hygiene objects, were more likely of having spontaneous resolution. HCV alternative routes of transmission, like sharing personal hygiene objects, are matter of controversy. Probably, small amounts of blood present in tooth brushes, shaving blades and cuticle nippers could explain HCV transmission in patient without other major risk factors. [18], [22], [39]–[42]. This hypothesis should be better investigated in future studies designed specifically to study HCV transmission routes.

In conclusion, HCV co-infection is frequent among patients with HIV infection in our State, and the genotype 1 prevails. A small proportion of patients present spontaneous resolution of the infection by HCV. The higher proportion of infection by the difficult-to-treat HCV genotype 1 has important consequences for the public health system.
